# Perioperative chemotherapy versus adjuvant chemotherapy treatment for resectable locally advanced gastric cancer: a retrospective cohort study

**DOI:** 10.1186/s40001-023-01400-3

**Published:** 2023-10-09

**Authors:** Pengfei Su, Lin Jiang, Yingjing Zhang, Tian Yu, Hongyun Huang, Moxi Chen, Can Cao, Weiming Kang, Yuqin Liu, Jianchun Yu

**Affiliations:** 1 Department of General Surgery, Peking Union Medical College Hospital, Chinese Academy of Medical Sciences and Peking Union Medical College, Beijing, 100730 China; 2 Department of Pathology, Institute of Basic Medical Sciences, Chinese Academy of Medical Sciences and Peking Union Medical College, Beijing, 100005 China

**Keywords:** Gastric cancer, Perioperative chemotherapy, Neoadjuvant chemotherapy, Adjuvant chemotherapy, Survival benefit

## Abstract

**Background:**

Neoadjuvant chemotherapy (NAC) is increasingly used in locally advanced gastric cancer (LAGC), but the clinical safety and efficacy are still controversial. This study aims to compare perioperative chemotherapy (PEC) with adjuvant chemotherapy (AC) for resectable LAGC.

**Methods:**

Patients who underwent D2 gastrectomy for resectable LAGC were retrospectively reviewed, and divided into NSA group (NAC plus surgery and AC) and SA group (surgery followed by AC). The baseline characteristics and perioperative data were compared. Survival analysis was based on Kaplan–Meier method. Multivariate analyses for prognostic factors were based on the Cox regression.

**Results:**

A total of 450 patients were eligible for this study. 218 patients received NAC plus surgery and AC, while 232 upfront surgery followed by AC. The baseline characteristics were comparable between the two groups. NSA group showed significant superiority in R0 resection rate (*P* = 0.014), excised tumor size (*P* = 0.038), and tumor downstage (all *P* < 0.001). NAC did not affect postoperative complications or AC-related grade 3/4 adverse events. Patients in NSA group achieved significantly longer OS (*P* = 0.021) and DFS (*P* = 0.002). The Cox regression model showed that NAC was independently associated with better OS (HR 0.245, *P* = 0.039) and DFS (HR 0.591, *P* = 0.031).

**Conclusions:**

Compared with SA, the administration of NSA was considered safe and feasible for achieving higher R0 resection rate without increasing the postoperative complications or AC-related grade 3/4 adverse events, and NAC was independently associated with better OS and DFS for resectable LAGC.

## Introduction

Gastric cancer (GC) is the fifth most frequently diagnosed malignancy, accounting for the third leading cause of cancer-related death worldwide [1]. Due to the lack of typical clinical symptoms in early GC, most patients have progressed to the advanced stage at the initial diagnosis [2]. Surgery has been regarded as the only potentially curative intervention for resectable locally advanced gastric cancer (LAGC). Nevertheless, even after curative resection combined with lymphadenectomy, the rates of local recurrence and distant metastasis are still high and the prognosis remains unfavorable [3]. Over the past few decades, curative gastrectomy followed by adjuvant chemotherapy (AC) has been confirmed to improve overall survival (OS) and disease-free survival (DFS) compared with surgery alone [4, 5]. Since the landmark MAGIC trial, multimodal therapeutic patterns, including neoadjuvant chemotherapy (NAC) and perioperative chemotherapy (PEC) have been introduced to complement the conventional AC following curative gastrectomy and become the appealing treatment options for the potential to further prolong the survival of patients with LAGC [6–8]. However, multimodal therapeutic recommendations differ between regions, and the consensus on the optimum strategy and sequence has not yet been reached. The standard strategy in Europe is PEC plus curative gastrectomy, based on the MAGIC trial [6], adjuvant chemoradiotherapy following surgery in the North America, based on the INT0116 trial [9, 10], whereas in Asia, it is AC following surgery based on the CLASSIC [4] and ACTS-GC trials [5].

Large tumor burden, metastatic lymph nodes and systemic micrometastasis are unfavorable factors for curative resection. In response, NAC has been investigated to diminish tumors, reduce metastatic lymph nodes and micrometastases so as to improve curative resection rate for LAGC [6, 11, 12]. Despite these theoretical advantages, there is still a lack of evidence to answer whether NAC can further improve the survival of LAGC patients on the basis of AC following curative gastrectomy. In addition, there is concern that NAC would result in higher risk of postoperative complications and adverse events occurring during AC than upfront surgery followed by AC. Unlike in Europe and the North America, there appears to be an underutilization of NAC in Asia.

Therefore, we conducted this retrospective study to compared the clinical safety and efficacy between PEC and AC strategy, with the primary aim to investigate whether the presence of NAC could further improve the long-term survival of LAGC patients on the basis of AC following curative gastrectomy. The secondary aim was to assess the impact of NAC on postoperative complications and adverse events occurring during AC.

## Materials and methods

### Patients selection

Patients who received treatment for gastric cancer between March 2012 and December 2016 at the department of general surgery of Peking Union Medical College Hospital were retrospectively reviewed from our prospectively collected database. Patients treated with neoadjuvant chemotherapy followed by surgery and adjuvant chemotherapy, or surgery followed by adjuvant chemotherapy were included while patients suffering from other synchronous and/or prior malignant tumor; receiving radiotherapy; lacking some information on diagnosis, therapy or evaluation were excluded. Finally, a total of 450 patients were included. This retrospective study was reviewed and approved by the Institutional Review Board of Peking Union Medical College Hospital. Written informed consent of each patient was obtained.

### Data elements

The demographic and clinicopathologic characteristics, including age, gender, initial body mass index (BMI), tumor location, tumor size, tumor differentiation, signet ring cell features, Lauren type, clinical T stage, clinical N status and clinical TNM stage (defined according to the 8th edition AJCC Staging Manual) were collected [13]. In addition, characteristics during and after treatment, including the regimen and cycle of NAC and AC, duration of operation, estimated blood loss, receipt of intraoperative blood transfusion, extent of resection, margin of resection, number of lymph nodes resected, size of excised tumor, status of lymphovascular invasion, pathological T stage, pathological N stage, distant metastasis and pathological TNM stage were collected. In the NSA group, patients received NAC followed by D2 gastrectomy and AC. We performed NAC based on the guidelines of the National Comprehensive Cancer Network (NCCN) and European Society for Medical Oncology (ESMO) [14, 15]. NAC was administered with the combination of platinum drugs and 5-fluorouracil, such as SOX (S-1 and oxaliplatin), XELOX (capecitabine and oxaliplatin) and FOLFOX6 (5-fluorouracil, leucovorin and oxaliplatin). Patients in NSA and SA group all received postoperative chemotherapy, of which the regimens were also based on platinum drugs and 5-Fluorouracil. Adverse events occurring during adjuvant chemotherapy were graded according to the National Cancer Institute Common Terminology Criteria for Adverse Events (NCI-CTCAE; version 4.0) [16].

### Follow-up and outcomes

All patients were required to visit the outpatient clinic at 3 months interval during the first 2 years after completing treatment, and 6 months interval thereafter for 3 years. After 5 years, consultation and follow-ups occurred once per year. The dates and events of relapse and death were collected from telephone interviews or electrical medical records. The primary end points were overall survival (OS) and disease-free survival (DFS). OS was defined as the intervals from the date of surgery to death from any cause. DFS was determined as the interval from the date of surgery to either the first relapse or death from any cause. The last follow-up date was September 2022. The secondary end points were postoperative complications and adverse events occurring during adjuvant chemotherapy of patients.

### Statistical analysis

Categorical variables were described as proportions and continuous variables were described as mean ± standard deviation. Categorical variables were analyzed by using the χ^2^ test or Fisher’s exact test, and continuous data were analyzed by using the Student’s t test or the Mann–Whitney U test. Survival curves for OS and DFS were evaluated using the Kaplan–Meier method, and the log-rank test was used to compare survival difference. The Cox regression analysis was adopted to assess the prognostic risk of demographic and clinicopathologic characteristics on OS and DFS, and the statistically significant factors from the univariate analysis were then taken into the multivariable analysis. Statistical analyses were performed using SPSS 22.0 (SPSS Inc., Chicago, IL, USA) and GraphPad Prism 8 (GraphPad Prism Software, Inc., San Diego, CA, USA), and *P* values < 0.05 was considered statistically significant.

## Results

### Baseline characteristics and cohort comparison

A total of 450 patients were eligible for this study. 218 of these patients underwent neoadjuvant chemotherapy followed by surgery and adjuvant chemotherapy (NSA group), while 232 underwent surgery followed by adjuvant chemotherapy (SA group). The baseline characteristics of these patients are summarized in Table 1. There was no significant difference between the two groups with regard to the distributions of age, gender, tumor location, differentiated degree, signet ring cell features, Lauren type, clinical T stage, N status, TNM stage, regimen and cycles of AC (all *P* > 0.05). Initial body mass index (BMI) and pre-therapy tumor size were comparable between the two groups. For regimens of NAC, 115 patients (52.8%) received SOX regimen, 76 (34.9%) received XELOX and 27 (12.4%) received FOLFOX6 regimen. NAC was performed 2 to 4 cycles in the group. No major adverse events related to NAC were noted and none of the patients required termination of therapy in the cohort due to NAC-related complications.

**Table 1:** Baseline characteristics of patients

Characteristics	Total	NSA group	SA group	
	*n* = 450	*n* = 218	*n* = 232	*P*-value
Age (years)				0.757
≥ 60	234 (52.0)	115 (52.8)	119 (51.3)	
< 60	216 (48.0)	103 (47.2)	113 (48.7)	
Gender				0.523
Male	345 (76.7)	170 (78.0)	175 (75.4)	
Female	105 (23.3)	48 (22.0)	57 (24.6)	
Initial BMI				0.628
Mean (SD)	23.3 (5.9)	23.6 (6.3)	23.1 (5.2)	
Tumor location				0.564
Gastric	373 (82.9)	183 (83.9)	190 (81.9)	
GEJ	77 (17.1)	35 (16.1)	42 (18.1)	
Pre-therapy tumor size (cm)				0.533
Mean (SD)	5.0 (2.2)	5.2 (2.1)	4.9 (2.4)	
Tumor differentiation				0.367
Well	45 (10.0)	23 (10.6)	22 (9.5)	
Moderate	130 (28.9)	69 (31.7)	61 (26.3)	
Poorly	275 (61.1)	126 (57.8)	149 (64.2)	
Signet ring cell	132 (29.3)	70 (32.1)	62 (26.7)	0.210
Lauren type				0.353
Intestinal	212 (47.1)	94 (43.1)	118 (50.9)	
Diffuse	124 (27.6)	67 (30.7)	57 (24.6)	
Mixed	82 (18.2)	42 (19.3)	40 (17.2)	
Unknown	32 (7.1)	15 (6.9)	17 (7.3)	
Clinical T stage				0.904
T2	54 (12.0)	25 (11.5)	29 (12.5)	
T3	146 (32.4)	72 (33.0)	74 (31.9)	
T4a	234 (52.0)	112 (51.4)	122 (52.6)	
T4b	16 (3.6)	9 (4.1)	7 (3.0)	
Clinical N status				0.572
N-negative	19 (4.2)	8 (3.7)	11 (4.7)	
N-positive	431 (95.8)	210 (96.3)	221 (95.3)	
Clinical TNM stage				0.847
IIA	54 (12.0)	25 (11.5)	29 (12.5)	
IIB	19 (4.2)	8 (3.7)	11 (4.7)	
III	361 (80.2)	176 (80.7)	185 (79.7)	
IVA	16 (3.6)	9 (4.1)	7 (3.0)	
NAC regimen				NA
SOX	NA	115 (52.8)	NA	
XELOX	NA	76 (34.9)	NA	
FOLFOX6	NA	27 (12.4)	NA	
No. of NAC cycles				NA
2	NA	35 (16.1)	NA	
3	NA	41 (18.8)	NA	
4	NA	142 (65.1)	NA	
AC regimen				0.231
SOX	256 (56.9)	120 (55.0)	136 (58.6)	
XELOX	122 (27.1)	68 (31.2)	54 (23.3)	
FOLFOX6	52 (11.6)	21 (9.6)	31 (13.4)	
S-1 single agent	20 (4.4)	9 (4.1)	11 (4.7)	
No. of AC cycles				0.177
< 5	155 (34.4)	82 (37.6)	73 (31.5)	
5–8	235 (52.2)	104 (47.7)	131 (56.5)	
> 8	60 (13.3)	32 (14.7)	28 (12.1)	

Values are presented as number (%) unless otherwise indicated. NSA: neoadjuvant chemotherapy plus surgery plus adjuvant chemotherapy; SA: surgery plus adjuvant chemotherapy; BMI: body mass index; SD: standard deviation; GEJ: gastroesophageal junction; NAC: neoadjuvant chemotherapy; AC: adjuvant chemotherapy

### Surgical and pathological results

For the analysis of surgical outcomes, patients in NSA group achieved higher R0 resection rate (95.4% vs 89.2%; *P* = 0.014) and smaller excised tumor size (3.9 ± 1.7 vs 5.3 ± 2.1; *P* = 0.038), while the duration of operation, the amount of estimated blood loss, the number of resected lymph nodes, the distributions of intraoperative blood transfusion and the extent of resection were comparable between the two groups (all *P* > 0.05) (Table 2). Less patients in NAS group had distant metastasis (6 of 218 patients vs 29 of 232 patients; *P* < 0.001), and there was a significant tumor downstaging in pathological T stage (*P* < 0.001), pathological N stage (*P* < 0.001) and pathological TNM stage (*P* < 0.001). Furthermore, patients in NSA group had a pathologic complete response rate of 8.3% (18 of 218 patients), indicating better tumor downstaging associated with NAC.

**Table 2:** Operative data and pathological results of patients

Characteristics	NSA group	SA group	*P*-value
	*n* = 218	*n* = 232	
Duration of operation			0.672
Mean (SD), min	229.3 (46.4)	242.1 (37.8)	
Estimated blood loss			0.374
Mean (SD), ml	248.3 (215.8)	267.6 (189.2)	
Intraoperative blood transfusion	35 (16.1)	41 (17.7)	0.647
Extent of resection			0.117
Distal subtotal	137 (62.8)	162 (69.8)	
Total	81 (37.2)	70 (30.2)	
Margin of resection			0.014
R0	208 (95.4)	207 (89.2)	
R1 or R2	10 (4.6)	25 (10.8)	
LNs resected			0.805
Mean (SD)	31 (10)	33 (15)	
Excised tumor size (cm)			0.038
Mean (SD)	3.9 (1.7)	5.3 (2.1)	
Lymphovascular invasion	59 (27.1)	75 (32.3)	0.222
Pathological T stage			< 0.001
T0	18 (8.3)	0 (0.0)	
T1	24 (11.0)	12 (5.2)	
T2	34 (15.6)	18 (7.8)	
T3	101 (46.3)	114 (49.1)	
T4a	28 (12.8)	45 (19.4)	
T4b	13 (6.0)	43 (18.5)	
Pathological N stage			< 0.001
N0	127 (58.3)	70 (30.2)	
N1	41 (18.8)	62 (26.7)	
N2	30 (13.8)	50 (21.6)	
N3a	16 (7.3)	31 (13.4)	
N3b	4 (1.8)	19 (8.2)	
Distant metastasis			< 0.001
M0	212 (97.2)	203 (87.5)	
M1	6 (2.8)	29 (12.5)	
Pathological TNM stage			< 0.001
0	18 (8.3)	0 (0.0)	
I	36 (16.5)	22 (9.5)	
II	80 (36.7)	49 (21.1)	
III	78 (35.8)	132 (56.9)	
IV	6 (2.8)	29 (12.5)	

Values are presented as number (%) unless otherwise indicated. NSA: neoadjuvant chemotherapy plus surgery plus adjuvant chemotherapy; SA: surgery plus adjuvant chemotherapy; LNs: lymph nodes; SD: standard deviation

### Postoperative outcomes and adverse events

As shown in Table 3, the recovery courses, including the mean postoperative hospital stay, time to first flatus, time to first liquid diet, and the postoperative blood transfusion rate were not significantly different between the two groups (all *P* > 0.05). Totally, the rate of postoperative complications was 24.8% and 19.8% for NSA and SA group (*P* = 0.207), respectively. As regards to each subtype of postoperative complications, including surgery-related and system-related complications, no statistical difference was observed between the two groups (all *P* > 0.05). Table 4 shows the adverse events (calculated using NCI-CTCAE; version 4.0) during postoperative adjuvant chemotherapy. The NSA group had a higher occurrence of anemia (38.5% vs 25.0%, *P* = 0.002), leukopenia/neutropenia (46.8% vs 31.0%, *P* = 0.001) and hand-foot syndrome (8.7% vs 4.3%, *P* = 0.057) of all grades than the SA group. However, the occurrence rates of grade 3/4 adverse events were comparable between the two groups (all *P* > 0.05).

**Table 3:** Postoperative outcomes and complications of patients

Characteristics	NSA group	SA group	*P*-value
	*n* = 218	*n* = 232	
Postoperative hospital stay			0.158
Mean (SD), d	11.9 (8.0)	10.6 (6.1)	
Postoperative blood transfusion	27 (12.4)	33 (14.2)	0.566
Time to first flatus			0.208
Mean (SD), d	3.73 (1.26)	3.46 (0.84)	
Time to first liquid diet			0.368
Mean (SD), d	4.24 (3.16)	4.16 (2.49)	
Abdominal infection	5 (2.3)	4 (1.7)	0.745
Anastomotic stenosis	2 (0.9)	1 (0.4)	0.613
Anastomotic leakage	11 (5.0)	14 (6.0)	0.647
Dumping syndrome	3 (1.4)	7 (3.0)	0.341
Delayed gastric emptying	22 (10.1)	18 (7.8)	0.385
Wound problem	8 (3.7)	6 (2.6)	0.508
Gastrointestinal bleeding	2 (0.9)	3 (1.3)	1.000
Pleural effusion	4 (1.8)	4 (1.7)	1.000
Pneumonia	6 (2.8)	9 (3.9)	0.506
Overall	54 (24.8)	46 (19.8)	0.207

Values are presented as number (%) unless otherwise indicated. NSA: neoadjuvant chemotherapy plus surgery plus adjuvant chemotherapy; SA: surgery plus adjuvant chemotherapy; SD: standard deviation

**Table 4:** Adverse events occurring during adjuvant chemotherapy

Characteristics	NSA group, *n* = 218		SA group, *n* = 232		*P*-value
	Any grade	Grade 3/4	Any grade	Grade 3/4	
Nausea/vomiting	64 (29.4)	13 (6.0)	57 (24.6)	20 (8.6)	^**a**^0.252; ^b^0.280
Diarrhea	21 (9.6)	4 (1.8)	30 (12.9)	7 (3.0)	^**a**^0.270; ^b^0.546
Constipation	16 (7.3)	1 (0.5)	21 (9.1)	2 (0.9)	^**a**^0.509; ^b^1.000
Anemia	84 (38.5)	21 (9.6)	58 (25.0)	30 (12.9)	^**a**^0.002; ^b^0.300
Leukopenia/neutropenia	102 (46.8)	29 (13.3)	72 (31.0)	34 (14.7)	^**a**^0.001; ^b^0.679
Thrombocytopenia	42 (19.3)	11 (5.0)	38 (16.4)	14 (6.0)	^**a**^0.423; ^b^0.647
Allergy	26 (11.9)	3 (1.4)	34 (14.7)	3 (1.3)	^**a**^0.395; ^b^1.000
Hand-foot syndrome	19 (8.7)	6 (2.8)	10 (4.3)	4 (1.7)	^**a**^0.057; ^b^0.533
Liver dysfunction	14 (6.4)	3 (1.4)	18 (7.8)	7 (3.0)	^**a**^0.581; ^b^0.341
Fever	21 (9.6)	6 (2.8)	19 (8.2)	5 (2.2)	^**a**^0.591; ^b^0.682
Urinary tract infection	9 (4.1)	3 (1.4)	11 (4.7)	2 (0.9)	^**a**^0.753; ^b^0.677

Values are presented as number (%). NSA: neoadjuvant chemotherapy plus surgery plus adjuvant chemotherapy; SA: surgery plus adjuvant chemotherapy. ^a^ for the statistic difference of any grade adverse events between the two groups. ^b^ for the statistic difference of grade 3/4 adverse events between the two groups

### Survival analyses

**Fig. 1:**
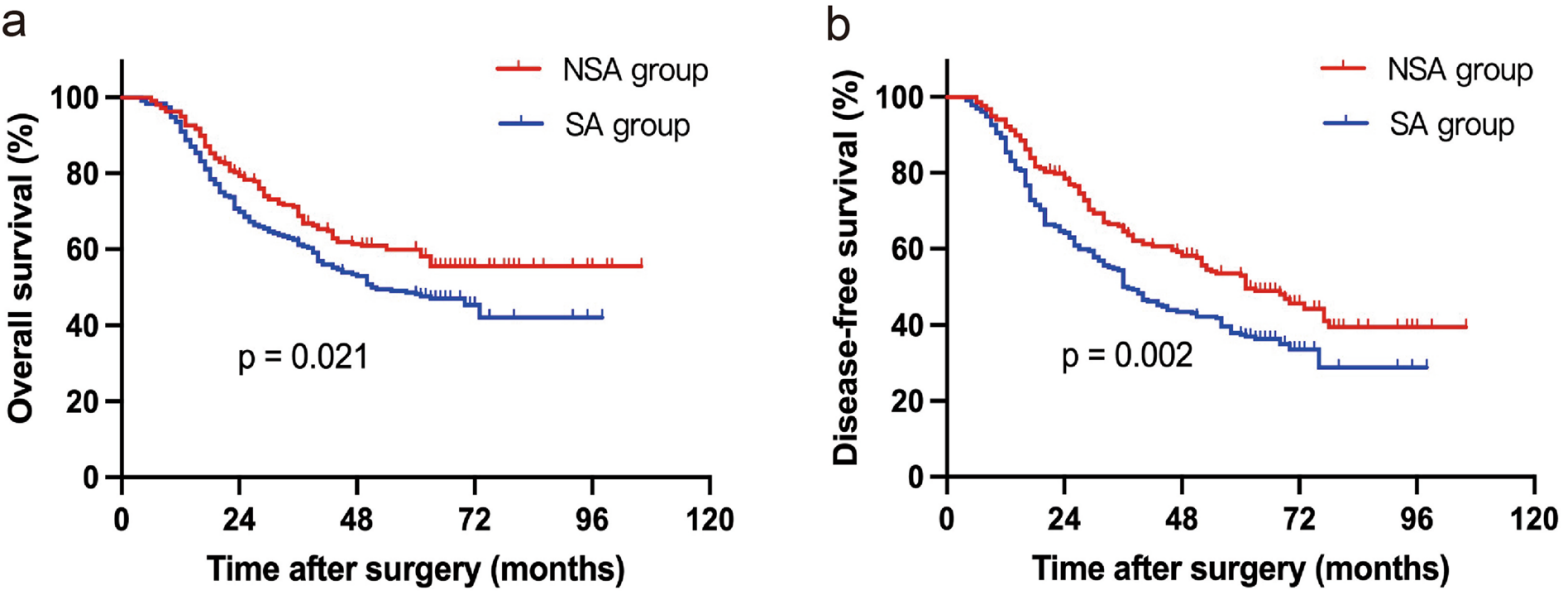
Survival of patients in the NSA and SA groups. **A** Kaplan–Meier survival curves for overall survival and **B** disease-free survival

The median follow‐up time for the 450 patients was 57.2 months (range, 4.1–106.3 months). Kaplan–Meier survival curves for OS and DFS are depicted in Fig. 1. Patients in NSA group showed a significantly longer OS and DFS than SA group (OS: *P* = 0.021; DFS: *P* = 0.002). The median OS in NSA group and SA group were undefined and 51.0 months with the 3-year OS rate of 68.8% and 61.2%, and the 5-year OS rate of 60.9% and 49.8%, respectively. In addition, patients in NSA group achieved an improvement in 3-year DFS (64.6% vs 58.0%) and 5-year DFS rate (58.2% vs 42.2%) compared with patients in SA group, and the median DFS was 61.0 and 36.5 months, respectively. Univariable Cox regression analysis revealed that pre-therapy tumor size, clinical TNM stage, NAC, pathological T stage, pathological N stage, lymphovascular invasion and pathological TNM stage were associated with OS and DFS (Table 5). Histological grade was associated with DFS but not OS. The multivariable analysis demonstrated that pre-therapy tumor size (HR [hazard ratio] 2.091, 95% CI 1.176–3.715, *P* = 0.012), NAC (HR 0.245, 95% CI 0.103–0.811, *P* = 0.039), pathological T stage (HR 2.266, 95% CI 0.997–3.124, *P* = 0.027) and pathological TNM stage (HR 1.992, 95% CI 1.067–3.736, *P* = 0.012) were independently predictive factors for OS, while pre-therapy tumor size (HR 1.821, 95% CI 1.050–3.156, *P* = 0.033), NAC (HR 0.591, 95% CI 0.364–0.598, *P* = 0.031), histological grade (HR 1.905, 95% CI 1.125–3.328, *P* = 0.046), lymphovascular invasion (HR 1.984, 95% CI 1.254–2.796, *P* = 0.025) and pathological TNM stage (HR 2.233, 95% CI 1.198–4.162, *P* = 0.011) were independently associated with DFS (Table 6).

**Table 5:** Univariable Cox regression analysis of factors associated with survival of patients

Variable	OS		DFS	
Univariable analysis	HR (95% CI)	*P*-value	HR (95% CI)	*P*-value
Age (years)				
≥ 60 vs. < 60	0.982 (0.510–1.894)	0.957	1.021 (0.533–1.957)	0.950
Gender				
Female vs. male	1.184 (0.625–2.245)	0.604	1.118 (0.590–2.116)	0.733
Pre-therapy tumor size (cm)				
≥ 5 vs. < 5	1.850 (1.121–3.055)	0.016	1.998 (1.048–3.809)	0.036
Tumor location				
Gastric vs. GEJ	1.085 (0.555–2.119)	0.812	1.388 (0.713–2.702)	0.335
Extent of resection				
Subtotal vs. total	1.216 (0.630–2.347)	0.560	1.682 (0.867–3.262)	0.124
Clinical T stage				
T3/4a/4b vs. T2	1.614 (0.847–3.076)	0.146	1.431 (0.724–2.827)	0.302
Clinical N stage				
N-positive vs. N-negative	1.309 (0.681–2.515)	0.420	1.690 (0.795–3.591)	0.173
Clinical TNM stage				
III/IVA vs. IIA/IIB	1.931 (1.007–3.700)	0.047	2.329 (1.107–4.899)	0.026
NAC				
Yes vs. no	0.437 (0.231–0.828)	0.011	0.382 (0.182–0.800)	0.008
Lauren type				
Intestinal vs. diffuse/mixed	0.884 (0.456–1.715)	0.715	0.6721 (0.469–1.073)	0.664
Histological grade				
Poorly vs. well/moderate	1.687 (0.856–3.726)	0.167	2.306 (1.090–4.878)	0.023
Signet ring cell				
Yes vs. no	1.461 (0.826–2.341)	0.097	1.304 (0.724–2.431)	0.137
Pathological T stage				
T3/4a/4b vs. T0/1/2	2.384 (1.219–4.661)	0.011	2.277 (1.028–5.040)	0.042
Pathological N stage				
N-positive vs. N-negative	1.944 (0.994–3.800)	0.052	2.049 (1.005–4.174)	0.048
Lymphovascular invasion				
Yes vs. no	2.153 (1.123–4.128)	0.021	2.600 (1.289–5.242)	0.008
Pathological TNM stage				
III/IV vs. 0/I/II	3.487 (1.810–6.717)	< 0.001	3.413 (1.723–6.761)	< 0.001

OS: overall survival; DFS: disease-free survival; HR: hazard ratio; CI: confidence interval; GEJ: gastroesophageal junction; NAC: neoadjuvant chemotherapy

**Table 6:** Multivariable Cox regression analysis of factors associated with survival of patients

Variable	OS		DFS	
**Multivariable analysis**	**HR (95% CI)**	***P*-value**	**HR (95% CI)**	***P*-value**
Pre-therapy tumor size (cm)				
≥ 5 vs. < 5	2.091 (1.176–3.715)	0.012	1.821 (1.050–3.156)	0.033
Clinical TNM stage				
III/IVA vs. IIA/IIB	1.488 (0.611–2.436)	0.275	1.628 (0.925–2.864)	0.091
NAC				
Yes vs. no	0.245 (0.103–0.811)	0.039	0.591 (0.364–0.958)	0.031
Histological grade				
Poorly vs. well/moderate	-	-	1.905 (1.125–3.328)	0.046
Pathological T stage				
T3/4a/4b vs. T0/1/2	2.266 (0.997–3.124)	0.027	1.607 (0.974–2.652)	0.063
Pathological N stage				
N-positive vs. N-negative	1.111 (0.677–1.821)	0.677	1.109 (0.599–2.052)	0.742
Lymphovascular invasion				
Yes vs. no	1.372 (0.917–1.821)	0.531	1.984 (1.254–2.796)	0.025
Pathological TNM stage				
III/IV vs. 0/I/II	1.992 (1.062–3.736)	0.012	2.233 (1.198–4.162)	0.011

OS overall survival; DFS disease-free survival; HR hazard ratio; CI confidence interval; NAC, neoadjuvant chemotherapy

## Discussion

Curative gastrectomy combined with D2 lymphadenectomy has been accepted as the main treatment choice for LAGC in both Asian and Western countries, whereas locoregional recurrence and systemic micrometastases seriously affected patients’ prognosis [17, 18]. In clinical practice, surgery alone can hardly achieve the radical cure for LAGC, even though the extended lymphadenectomy is performed. Therefore, the key to prolonging survival is to improve the R0 resection rate, reduce the rate of local recurrence and distant metastasis. Evidence-based multiple treatment modalities, combining surgery with neoadjuvant and/or adjuvant chemotherapy, have been generally applied in the treatment of LAGC and the prognosis of patients was improved during the past decades [4–6, 19]. As mentioned above, perioperative chemotherapy or postoperative chemoradiotherapy has been the standard treatment for LAGC in Western countries, while postoperative chemotherapy was preferred in Asia [4–6, 9, 10]. The comparison of these treatment modalities remained controversial, there has not yet been global consensus on the appropriate patients with LAGC who should receive perioperative chemotherapy and the superiority of perioperative chemotherapy is still being explored.

The MAGIC and FNLCC/FFCD trials have confirmed the superiority of perioperative chemotherapy over surgery alone, with higher R0 resection and pCR rates and better OS, even though fewer than 50% patients completed the postoperative chemotherapy and a subset of patients were actually diagnosed with gastroesophageal junction tumor in these two trials [6, 11]. As a part of perioperative chemotherapy, the value of neoadjuvant chemotherapy in improving the prognosis of LAGC patients has not yet been well illustrated. A network meta-analysis of 33 randomized controlled trials demonstrated that perioperative chemotherapy had survival advantage over adjuvant therapy in patients with operable gastric cancer [20]. However, more high-quality data are requisite to verify this concept. The RESOLVE study, a phase 3 randomised controlled trial (RCT), showed that perioperative chemotherapy could increase the 3-year DFS rate by approximate 8.3% compared with adjuvant chemotherapy alone [21]. This RCT mainly recruited patients with relatively late stage cT4aN + M0 or T4bNanyM0 disease, a relatively narrow range of indication. Another phase 3 study, the PRODIGY study, drew a conclusion that perioperative chemotherapy could significantly improve the 3-year PFS, whereas the 3-year OS of patients received perioperative chemotherapy was comparable with that received adjuvant chemotherapy [22]. Despite the increasing use of neoadjuvant chemotherapy after MAGIC trial, the main treatment modality for LAGC still remains surgery. No global agreement on the appropriate population of patients with LAGC who should receive perioperative chemotherapy, the challenge in precise staging and the perioperative complications and chemotherapy-related adverse events might be the major reasons.

At present study, a total of 450 patients with cT2-4bNanyM0 stage met the inclusion criteria. All patients underwent D2 gastrectomy followed by adjuvant chemotherapy, and patients were divided into the NSA group and SA group, according to whether they have received neoadjuvant chemotherapy or not. There was no significant difference in the baseline characteristics between the two groups. Consistent with the previous results of several studies, our results suggested a superior tumor downstage rate in NSA group [12, 21, 23, 24]. Patients in NSA group were less likely to have pT3-4bN + disease, the pTNM stage was lower and the R0 resection rate was higher compared with SA group. Additionally, being different from that the pre-therapy tumor size was comparable among patients who received neoadjuvant chemotherapy or not, the excised tumor was smaller in NSA group. Survival analysis showed that the 5-year OS and DFS rates of patients in the NSA group were significantly higher than those in the SA group. We consider that the difference in survival between the two groups was due to whether NAC was used. Moreover, the 3-year DFS rate for patients in the NSA group was similar to the results in the RESOLVE [21] and PRODIGY [22] studies (64.6% vs 62.0 vs 66.3%). However, the 5-year OS rate for patients in the NSA group was higher than that in the MAGIC trial [6] and FNLCC/FFCD trial [11] (60.9% vs 36% vs 38%), for which the main reason may be the much higher R0 resection rate (95.4% vs 69% vs 84%).

One of the major arguments with the use of PEC is that it might increase the perioperative complications and chemotherapy-related adverse events [25]. In the present study, no major adverse events related to NAC were noted and none of the patients required termination of therapy due to NAC-related complications. Although higher occurrence of anemia, leukopenia/neutropenia and hand-foot syndrome of all grades were recorded in NSA group, the rate of grade 3/4 adverse events was comparable between the two groups during postoperative adjuvant chemotherapy, and no death events occurred. Moreover, patients may suffer from post-gastrectomy complications, such as anastomotic stenosis, dumping syndrome, delayed gastric emptying or anemia, all of which can delay the commencement of postoperative adjuvant chemotherapy [26]. It was worth noting that there was no significant difference in postoperative outcomes and complications between the two groups. This result further confirmed the safety of the perioperative treatment pattern.

There have been currently no unified standard indications for the application of neoadjuvant chemotherapy in LAGC. The Japan Clinical Oncology Group suggested that LAGC patients with clinical T3/T4 and cN+ stage were suitable to receive neoadjuvant chemotherapy [27]. The indications of neoadjuvant chemotherapy for GC in the 2021 Chinese Society of Clinical Oncology (CSCO) guidelines were patients with clinical staging T3–4a and N+ stage [28]; whereas, the ESMO clinical practice guidelines recommended a wider range of indications for neoadjuvant chemotherapy (> cT1N0) [29]. Survival benefits might be brought to patients in the condition of formulating suitable criteria to select the right people and using individualized and suitable treatment. In addition, well-designed studies are required to explore effective chemotherapy regimens and cycles. Precise staging and timely identification of the pathological response would lead to either an intensification of the neoadjuvant strategy in responding patients or to consider surgical treatment in the absence of clinical benefit.

We acknowledge that the present study contains certain limitations. Due to its retrospective nature and relatively limited number of patients at a single institution, potential selection bias and excessive hazard ratios in the analysis might exist. Second, even chemotherapy regimen was basically based on platinum drugs and 5-fluorouracil regimens, it was not standardized for NAC or AC, the effects of different regimens were not analyzed. Third, the patient cohort is a selected group (all had undergone resection and AC), and thus, the conclusion could not be extrapolated to all LAGC patients. Finally, our follow-up was relatively short. Despite the limitations above, the present study verified the superiority of perioperative chemotherapy for Asian patients with LAGC to a certain extent.

## Conclusions

Compared with SA, the administration of NSA was considered safe and feasible for achieving higher R0 resection rate without increasing the postoperative complications or AC-related grade 3/4 adverse events, and NAC was independently associated with better OS and DFS for resectable LAGC. Our findings are expected to be supported by more high-quality prospective data.

## Data Availability

The datasets generated during and/or analysed during the current study are available from the corresponding author on reasonable request.
